# A multisectoral approach to advance health equity in rural northern Arizona: county-level leaders’ perspectives on health equity

**DOI:** 10.1186/s12889-022-13279-6

**Published:** 2022-05-13

**Authors:** Dulce J. Jiménez, Samantha Sabo, Mark Remiker, Melinda Smith, Alexandra E. Samarron Longorio, Heather J. Williamson, Carmenlita Chief, Nicolette I. Teufel-Shone

**Affiliations:** Center for Health Equity Research, Northern Arizona University, PO Box 4065, Flagstaff, AZ 86011 USA

**Keywords:** Health equity, Multisectoral, Community-engaged, Social determinants of health, Collective impact

## Abstract

**Background:**

Multisectoral and public–private partnerships are critical in building the necessary infrastructure, policy, and political will to ameliorate health inequity. A focus on health equity by researchers, practitioners, and decision-makers prioritizes action to address the systematic, avoidable, and unjust differences in health status across population groups sustained over time and generations that are beyond the control of individuals. Health equity requires a collective process in shaping the health and wellbeing of the communities in which we live, learn, work, play, and grow. This paper explores multisectoral leaders’ understanding of the social, environmental, and economic conditions that produce and sustain health inequity in northern Arizona, a geographically expansive, largely rural, and culturally diverse region.

**Methods:**

Data are drawn from the Southwest Health Equity Research Collaborative’s Regional Health Equity Survey (RHES). The RHES is a community-engaged, cross-sectional online survey comprised of 31 close-ended and 17 open-ended questions. Created to assess cross-sectoral regional and collective capacity to address health inequity and inform multisectoral action for improving community health, the RHES targeted leaders representing five rural northern Arizona counties and 13 sectors. Select open-ended questions were analyzed using an a priori coding scheme and emergent coding with thematic analysis.

**Results:**

Although leaders were provided the definition and asked to describe the root causes of inequities, the majority of leaders described social determinants of health (SDoH). When leaders described root causes of health inequity, they articulated systemic factors affecting their communities, describing discrimination and unequal allocation of power and resources. Most leaders described the SDoH by discussing compounding factors of poverty, transportation, housing, and rurality among others, that together exacerbate inequity. Leaders also identified specific strategies to address SDoH and advance health equity in their communities, ranging from providing direct services to activating partnerships across organizations and sectors in advocacy for policy change.

**Conclusion:**

Our findings indicate that community leaders in the northern Arizona region acknowledge the importance of multisectoral collaborations in improving health equity for the populations that they serve. However, a common understanding of health equity remains to be widely established, which is essential for conducting effective multisectoral work to advance health equity.

**Supplementary Information:**

The online version contains supplementary material available at 10.1186/s12889-022-13279-6.

## Introduction

In the last decade, public health researchers and practitioners have shifted from identifying health disparities to tackling the economic, social, and environmental root causes of health inequity [1, 2]. This movement was motivated by the recognition of significant differences in the burden of disease in communities of color and the change required in addressing the larger patterns of social inequalities that produce health inequities. To approach this fundamental issue, the social determinants of health (SDoH) framework has emerged to define how the conditions in which people are born, grow, live, and, work contribute to sickness and wellness [3]. While an important contribution to public health, the SDoH framework does not explicitly address the underlying social and institutional inequities largely based on class, race, disability status, citizenship, and gender. That is to say, unlike an equity lens, a SDoH framework does not offer a critical analysis of the inequitable distribution of power and resources, and the institutional policies and practices that influence opportunities to be healthy. Health equity offers a framework of knowledge and practice rooted in a commitment to reducing and ultimately eliminating health disparities and addressing the SDoH [4, 5]. The shift in lexicon, principles, and practice from health disparities, to the SDoH and finally to an intentional commitment to health equity, signals a paradigm shift rooted in a critical understanding of justice, fairness, and power structures [4]. While health equity frameworks are more common within the public health and health care sectors, other sectors critical to advancing health equity, such as housing, economic development, transportation, education, and justice, among many others, have yet to be widely engaged.

Collaboration between diverse sectors, including public, private, and grassroots organizations, with a shared goal of achieving health equity, can be defined as a multisectoral approach to health equity [6]. This collaborative approach was highlighted in the seminal 2017 National Academies of Sciences, Engineering, and Medicine *Communities in Action: Pathways to Health Equity* [3], which described community-driven health equity projects that involved two or more sectors and emphasized the “pathways” in which diverse sectors, such as civil rights, business, education, and transportation could promote health equity through programming and policy [3]. A multisectoral approach involves bringing people from different sectors together to strategize around a common goal.

Furthermore, the value and potential impact of multisectoral work is increasingly recognized on a global scale. The United Nation’s 2030 Agenda for Sustainable Development provides a plan of action with 17 goals for human development, prosperity, and peace, and the planet that aims to leave no one behind [7]. Goal 3 (Ensure healthy lives and promote wellbeing for all at all ages) cannot be achieved without mobilizing a multisectoral approach [7]. Multisectoral policy and action, which employs evidence-based policies and actions to systematically address social, economic, and environmental determinants of health and individual behavior across all sectors, is one of three inter-related primary components of the World Health Organization’s (WHO) and the United Nations Children’s Fund’s (UNICEF) Operational Framework for Primary Health Care [8]. The importance of multisectoral partnerships is further highlighted in the Declaration of Astana on Primary Health Care, which was released in 2018 and represents a recommitment to strengthening primary health care, addressing gaps in access to services across the SDoH, and advancing health equity across the world [9].

Multisectoral health equity approaches come with challenges, for example, aligning the interests of different stakeholders [10], developing multisectoral partnerships that are sustainable [10–13], and the time required for relationship building [11]. Despite these challenges, a multisectoral approach is identified as an effective means to address health inequities, align public health priorities across sectors and with the community, and improve the efficiency of public health efforts [6, 11, 14–16]. However, the lack of a common language and clarity around the concept of health equity remains a barrier to forming and sustaining multisectoral partnerships with the goal of advancing health equity [17, 18]. Without a shared understanding of health equity, partners from different sectors may struggle to participate in important conversations, develop policy and practice goals, and allocate resources to address health inequities [17, 18].

To explore this issue in northern Arizona, the present paper documents the understanding of the SDoH and strategies to address health inequities both locally and regionally from the perspective of leaders representing various sectors beyond public health and health care. Northern Arizona is a geographically expansive (over 6,000 square miles of land and home to 12 federally recognized American Indian tribes), largely rural (37% of residents live in areas with a population of fewer than 2500 people), and culturally diverse (62.5% White, 22.5% American Indian, and 11% Hispanic) region, making it a scientifically significant region for a focus on health equity issues [19].

## Methods

Data are drawn from an effort of the Community Engagement Core (CEC) of Northern Arizona University’s Southwest Health Equity Research Collaborative (SHERC), a Research Center in Minority Institutions (RCMI) funded by the National Institutes of Health (NIH), National Institute of Minority Health and Health Disparities (NIMHD). The CEC is guided by asset-based, community-engaged frameworks that recognize health as a product of multiple social determinants and inequities driven by systems of poverty, structural racism, ableism, misogyny, and discrimination, in which community-based solutions are essential yet insufficient alone to achieve health equity [3, 20].

Beginning in 2018, the SHERC CEC engaged county-level leaders from various sectors to identify drivers of health inequity and identify local assets nurturing multisectoral approaches to addressing the root causes of inequity in the region. The CEC developed the 2019 Regional Health Equity Survey (RHES), a cross-sectional online survey designed to explore how leaders and decision-makers understand and describe health inequity and strategize to address health inequities in their communities (for the complete survey, see Additional file 1, *RHES-Data Collection Instrument*). The survey was administered to leaders in five counties and 13 sectors in northern Arizona. Development of the RHES was guided by a regional, multisectoral community advisory council (CAC) of 11 personally, professionally, and geographically diverse members that assisted in design and implementation. The CAC comprised northern Arizonan leaders from different sectors important to advancing health equity, such as public health, education, early childhood development, criminal justice, and policy. CAC members also represented the vast geographic expanse and cultural diversity of the region [21].

### Regional Health Equity Survey (RHES)

The CEC engaged members of the CAC to generate specific survey constructs important to achieving, maintaining, and scaling health equity in the region. Additionally, the RHES is adapted from the Bay Area Regional Health Inequities Initiative’s (BARHII) Organizational Self-Assessment for Addressing Health Inequities Toolkit [22], which helps public health leaders identify the skills, organizational practices, and infrastructure needed to take action in addressing health equity. CAC members and SHERC staff engaged in two rounds of edits and through a process of consensus finalized the RHES, comprised of 48 questions, including 17 open-ended questions. Constructs include the distribution of resources in the communities served, personal understanding of SDoH, organizational capacity to address health inequities, extent and focus of cross-sectoral partnerships, data use in decision-making, the role of research in addressing health inequities in the community, and priority areas for future research. A set of close and open-ended questions were used to characterize multisectoral leaders’ understanding of the following: (1) the primary community they serve, including how resources are distributed within the community; (2) the SDoH and the root causes of health inequity; and (3) strategies to address health equity locally and regionally.

### Participants

The population surveyed by the RHES included community, organizational, and grassroots leaders from five northern Arizona counties, representative of the following sectors: 1) community health and economic development; 2) health and human services; 3) law, justice, and public safety; 4) parks and recreation; 5) policy; 6) early childhood development; 7) transportation; 8) food systems; 9) housing; 10) education; 11) arts, music, and culture; 12) planning and zoning; and 13) cultural resources management. Potential participants for the RHES were identified in three ways: 1) extensive internet searches targeting individuals in organizational leadership positions across the 13 sectors and five counties of northern Arizona; 2) CAC members nominated leaders from their sectors and regions; 3) CEC staff presented the survey and distributed sign-up sheets at county-level leadership meetings involving the target participants. Names of all potential participants were compiled, duplicates were deleted, and participant lists were created for each county across sectors. Two to three county champions per county vetted each county’s list, removing individuals who were no longer in those positions and adding names in sectors where representation was absent. County champions were invited based on county leadership positions (e.g., assistant county manager and local public health director) and were not compensated.

Finalized participant lists were used by county champions to introduce the RHES to all potential participants and alert them of the survey administration plans. Invitations to participate in the RHES, including links to the survey, were circulated electronically by CEC staff one day after introductory e-mails were sent. Two reminder e-mails were sent to participants 2 and 4 weeks after the initial invitation. A US $25 gift card was offered as compensation to all respondents for their participation. The RHES was distributed via e-mail using an online survey software (Qualtrics XM, Provo, UT). The RHES was reviewed and approved by the Northern Arizona University Institutional Review Board (project number: 1198096–1). Detailed methods used to develop and implement the RHES are described in Remiker (2021) [19].

### Analysis

All descriptive statistics were analyzed using IBM Statistical Package for Social Sciences software (version 26). Considering the differences in types of responses from each open-ended question, qualitative data were analyzed using either a priori coding or emergent coding and a thematic analysis approach in ATLAS.ti 8 (ATLAS.ti Scientific Software Development GmbH, Berlin, Germany).

To analyze the open-ended question exploring SDoH and root causes, BARHII definitions were used as an initial a priori broad coding scheme to understand when participants were discussing SDoH versus root causes of health inequity (Table 1). The Vitalyst Health Foundation’s *Elements of a Healthy Community* [23] were then applied to the a priori codebook to include specific SDoH. To capture the full range of SDoH described by participants, codes that emerged from the data were also added to the a priori codebook. The full data set was independently coded by one researcher. Then, a second researcher independently coded 25% of the responses. The two researchers reviewed their independent coding together until consensus on codes and themes was achieved through intensive discussion. As a final step, the full data set was reviewed and revised for accurate coding by the first researcher based on the consensus decisions [24].

**Table 1:** Regional Health Equity Survey Thematic Code Definitions [22]

Broad Code	Definition
**Health Inequity**	Health inequities are the systematic, avoidable, unfair, and unjust differences in health status across population groups. These inequities are sustained overtime and generations and are beyond the control of individuals. These differences follow the larger patterns of inequality that exist in society. This is different from the term health disparities, which emphasizes that differences exist, but does not consider their relationship patterns of social inequalities.
**Root Causes of Health Inequity**	The root causes of health inequity are the underlying social, economic, and environmental inequalities which create different living conditions. Discrimination based on class, race, ethnicity, immigration status, gender, sexual orientation, disability and other ‘isms’ influence the distribution of resources and power. Past discriminatory practices are reinforced in the policies and practices of institutions that define the context of our daily lives. This in turn creates an unequal distribution of beneficial opportunities and negative exposures, resulting in health inequities.
**Social Determinant of Health (SDoH)**	The social determinants of health are the conditions in which people are born, grow, live, work and age (e.g. air quality, schools, parks, jobs, and housing conditions etc.). This term does not address how or why these social, economic, and environmental conditions are inequitably distributed throughout society.

The remaining open-ended question (i.e., strategies to address health equity) was analyzed using emergent coding, where one researcher read through all the responses, summarized broad themes, and shared findings with a second researcher. During a collective review of participants’ summarized responses, codes and more specific themes that emerged from the data were discussed and agreed upon. The first researcher then applied the refined codes to the data and, together with the second researcher, achieved consensus on the final themes.

## Results

A total of 206 of the 560 invited multisectoral representatives (response rate 37%) from northern Arizona participated in the RHES. Of those who participated, 64% (132/206) completed the entire survey, responding to the open-ended qualitative questions of interest about: (1) the root causes of health inequity that impact the health of the community they serve, and (2) strategies to address health equity. Among those that answered open-ended questions, half held government positions at the federal, state, county, and municipality level and one-third worked for non-government organizations, including community-based organizations, community groups or coalitions, faith-based organizations, and non-profits. Participants reported holding leadership positions such as county managers and department directors, chief of police, superintendents, presidents, CEOs, and executive directors. The demographic breakdown of all survey participants did not significantly differ from those that provided qualitative responses. Therefore, Table 2 provides participant demographics for the entire survey reflective of qualitative respondents.

**Table 2:** Participant demographics

Demographic Characteristic	Total Participants N (%)
**Sex**	***N* = 129**
Male	56 (43.4)
Female	69 (53.5)
Other	1 (0.8)
Prefer not to answer	3 (2.3)
**Race and Ethnicity**	***N* = 129**
American Indian/Alaskan Native	3 (2.3)
Asian/Pacific Islander	1 (0.8)
Black/African American	3 (2.3)
Hispanic/Latino	6 (4.7)
White	108 (83.7)
Other	3 (2.3)
Prefer not to answer	5 (3.9)
**Age in years**	***N* = 127**
Mean (SD)	49 (11.6)
**Position time in years**	***N* = 195**
Mean (SD)	5.3 (6.0)
**Sector time in years**	***N* = 194**
Mean (SD)	16.6 (11.1)
**County**	***N* = 206**
Apache	8 (3.9)
Coconino	94 (45.6)
Mohave	34 (16.5)
Navajo	28 (13.6)
Yavapai	42 (20.4)
**Organization**	***N* = 204**
Government	102 (49.5)
Non-government	57 (27.7)
Private	11 (5.3)
Academic	20 (9.7)
Other	14 (6.8)
**Work Directly with Community Constituents**	***N* = 192**
Yes	37 (19.3)
No	155 (80.7)

Participants were primarily middle-aged, predominantly white leaders, with slightly more female participation compared to males. Leaders were well established within their sectors with an average of 16 years in the field and stable in their positions with an average of five years in their current leadership role. All participants held leadership positions and largely reported they did not work directly with the community in their current roles.

**Fig. 1:**
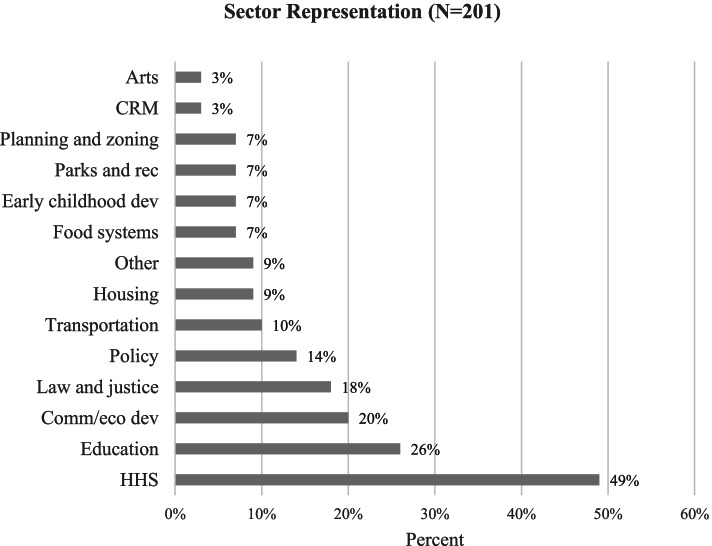
Sector Representation. Note: Sector respondents were allowed to check all that apply, “CRM” = Cultural Resource Management, “HHS” = Health and human services, “Comm./eco dev” = Community and Economic Development

Participants could identify with more than one sector. While all 13 sectors were represented, 95% of all participants identified with either health and human services (49%), education (26%), or community and economic development (20%) (Fig. 1).

### Community demographics

Two questions elicited characteristics of the communities served, including survey questions regarding leaders’ perceived understanding of the distribution of resources and services within the community they serve and an open-ended question asking respondents to describe the root causes of health inequity.

**Fig. 2:**
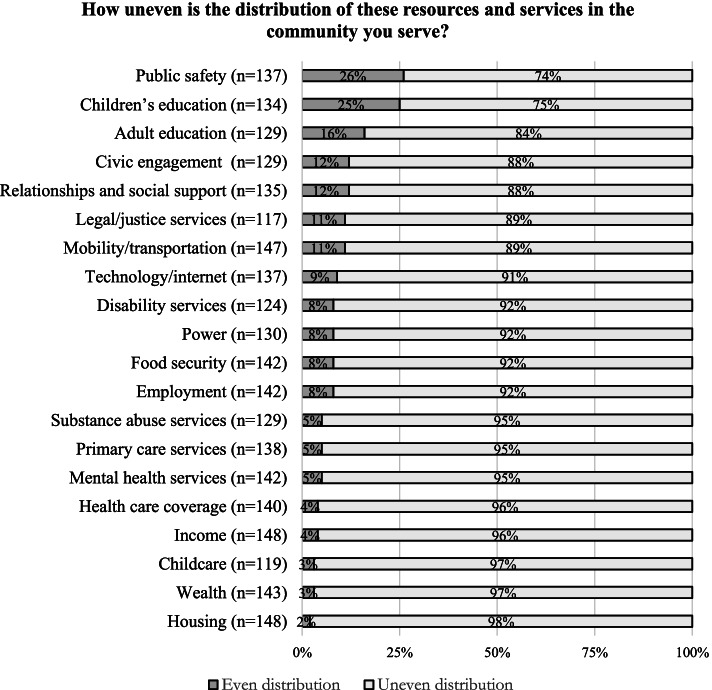
Perceived Distribution of Community Resources. Note: “Uneven distribution” includes responses to both “Very uneven” and “Somewhat uneven”

Upwards of 75% of participants reported resources and services to be unevenly or inequitably distributed across all sectors in the communities they serve (Fig. 2). According to one-quarter of survey participants, resources and services related to public safety and children’s education were perceived to be the most evenly or equitably distributed resources in the community.

### Multisectoral leaders' perceptions of health inequity

Participants were provided the definitions of the SDoH and root causes of health inequity outlined in Table 1 and asked to describe the root causes of health inequity in their community. Approximately 64% (*n* = 132) of participants responded to this question, and of those respondents, 11.4% (*n* = 15) provided a description that met the a priori definition of root causes of health inequity. The remaining participants provided an explanation that met the a priori definition of a SDoH and, in certain instances, leaders discussed other factors outside of the definitions applied for health inequity and SDoH. Exemplary quotes below are followed by the participant’s self-identified position and sector.

#### Root causes of health inequity

When leaders described root causes of inequity, they articulated systemic factors affecting the communities they serve and primarily described discrimination and unequal allocation of power and resources.

Some of the participants discussed the role discrimination plays in health and economic inequities in their communities. A few mentioned the type of discrimination, for example, based on race, sex, or class. Often, participants discussed discrimination and racism at both institutional and systemic levels and included perspectives on the deleterious effects of past and current policies perceived to be discriminatory, as articulated here:

“The root cause of health inequity is racism, systemic and institutional racism.” [Program manager, health and human services]

“The primary social conditions that impact the (housing and homeless) community I serve seem to be systemic racism and systemic poverty, which are, of course, inextricably related.” [Owner and research scientist, multisector]

“Many unincorporated townships passing laws stating the outright ban of “box stores” and other affordable/accessible services. Past policies around land distribution and land use disproportionately impacting Native communities. Infrastructure, or lack thereof, favoring higher income brackets and more able-bodied peoples: lack of sidewalks, elevators, handicap access, specialized services, etc. Classism affecting poor families, and especially families of color with childcare and early education opportunities being too expensive for most to afford. Free or reduced-price options fill up quickly with wait times being years long.” [Senior program coordinator, multisector]

Some participants described unequal allocation of power and resources. Most leaders who identified this phenomenon as a root cause of health inequity in their community gave robust explanations, providing examples of how this unequal allocation manifests as a complex interlocking of systems of power. Many leaders went on to describe how this contributes to inequity across SDoH and places specific communities, especially communities of color and people living in poverty, at a direct disadvantage. A regional director that identified as multisectoral and serves families with young children and higher risk populations identified root causes of health inequity and resulting effects as:

“The root cause here is the same as it is anywhere - unequal distribution of money, opportunity and power. How that shows up in my community is: Essential services provided in population hubs where cost of living is too high for those who most need services. Virtually no public transportation, wage disparity, lack of entry level employment opportunities, social and geographic isolation, technology vacuums outside of population hubs - although about 95% of the population owns a smart phone, data services for their use is too expensive, or there is spotty/no service in many of the outlying rural areas. Very limited affordable housing. The most "affordable" housing is the furthest from services/food/socialization. Yavapai (county) has been identified as a mental health desert. Yavapai (county) has been identified as a food desert. Limited access to quality medical specialists. Not enough medical providers. Very limited services for families with children with special needs.”

#### Social determinants of health

Approximately 75% of responses were categorized by the a priori definition of the SDoH. Guided by the SDoH specific code definition, we further categorized the types of SDoH leaders perceived as impacting the health of the community they serve. Based on these SDoH definitions, the predominant SDoH described by multisectoral leaders included economic opportunity, access to care, social and cultural cohesion, educational opportunity, transportation, housing, community safety, social justice, quality affordable food, environmental quality, and community design. These themes are summarized in Table 3 in order of frequency.

**Table 3:** Description of Themes for Social Determinants of Health Codes in Order of Frequency

Code	Theme	Exemplar Quotes
Economic Opportunity (*N* = 90)	Themes included poverty, income inequality, high cost of living, unemployment, limited job opportunities, limited high-quality job opportunities, and struggling economies locally and regionally. Economic opportunity was often viewed as inextricably linked with other factors such as healthcare, education, and secure housing. Access to quality, high-speed internet impacted connectivity and access to services.	“We don't have a consistent permanent employment base that keeps our community growing, competitive, or sustainable. There is a lack a critical infrastructure (water, internet, educated human capital work force) that would entice industry or business to move here creating employment growth.” [Fire chief, health and human services] “Lack of affordable housing, lack of high paying jobs, lack of jobs that offer healthcare and other benefits to employees, lack of medical providers.” [Economic development coordinator, community and economic development]
Access to Care (*N* = 51)	Leaders described a lack of healthcare providers and healthcare services within their communities, particularly as compounded by rurality. Healthcare costs, insurance status and coverage, and distance to health services were stated as potential challenges to access to care. A small handful of the participants mentioned an unequal distribution of health services in the communities they serve but did not further elaborate on why that inequality may exist.	“The root causes of health inequity in our area are due to limited rural supportive of services with access to care for families/individuals in need. Often times barriers include education of services, transportation, and financial support.” [Community impact director, multisector] “Poverty, distances people need to travel to health practitioners or facilities. Difficulty to attract health professionals to the area. Lack of access to specialized care, i.e. speech therapists, oncologists. Language barriers for the Native American elderly, and many reside in areas without running water or electricity. High level of addictions and social problems exceeds the practitioners and facilities that can intervene with the care needed.” [Library district director, multisector]
Social and Cultural Cohesion (*N* = 34)	Participants described a lack of support for mental health and lack of support systems and supportive relationships, families, and homes. Social and cultural cohesion was linked to or contributed to high rates of poor mental health, abuse, and childhood or family trauma, substance use, and stigma related to substance use and other health conditions like HIV. Fewer described topics such as language barriers and lack of opportunities to be engaged in the community. Both social and physical isolation were considered a function of rurality and the unique challenges rural communities face, including limited services and resources and lack of connection across sectors.	“High proportion of low-income jobs. High proportion of jobs without benefits. Use of illegal substances disproportionate to the size of the population. Children at high risk due to early traumas and dysfunctional families. Distance to specialized medical services.” [Superintendent, education] “My community has a higher cost of living with limited job opportunities and low pay. The youth lack support services and safe places to go. Community resources are limited and mental health resources are lacking.” [Health educator, multisector]
Educational Opportunity (*N* = 31)	“Lack of education” or just “education” were commonly listed with no further explanations. Some of the participants noted the lack of secondary education opportunities and support in their communities. Multiple participants also mentioned that low education status and low education standards are interlinked with the low employment within their communities.	“A lack of the education necessary to obtain a long term career.” [Regional director, early childhood development] “Lack of education, lack of money to attend school after free public education, lack of role models, lack of teamwork / communication for benefits of community, poor food options at the local store (soda, chips, candy), unhealthy lifestyle decisions.” [Superintendent, education]
Transportation (*N* = 26)	A lack of transportation (both personal and public transportation) was a major issue in leaders' communities. Multiple participants mentioned distance to services and rurality being contributing factors in transportation issues. Some tied their community’s transportation challenges to issues with the ability to work and to access food and health services.	“Much of our ridership is comprised of seniors, the disabled, tribal residents and low income families dependent on public transit to travel to medical appointments, the pharmacy and grocery store and government services. Public transportation is primary in alleviating one of the major root causes of health inequity; without a reliable mobility service, the health and welfare of our communities are significantly impacted.” [Grants and transit manager, transportation] “Economics and the high cost of living also many of the residents live out in the country where transportation is difficult.” [Public defender, law, justice, and public safety]
Housing (*N* = 24)	Housing concerns included lack of adequate and affordable housing, both to rent and buy, limited housing options, and homelessness in leaders' communities. Housing was often compounded by other factors, particularly by a lack of economic opportunities.	“We have relatively low unemployment but do have a lot of low income citizens. Affordable housing seems to be a big cause of some of our issues. People have to pay more towards their living conditions so takes away from other issues such as health care. [Director, health and human services] “Lack of affordable housing, transient population, inability to maintain job security.” [CEO, health and human services]
Community Safety (*N* = 16)	Leaders discussed issues such as domestic violence, child abuse, felony convictions, and substance (drugs and alcohol) abuse. Participants also mentioned homelessness, mental health challenges, and a lack of safe places for youth.	“Mental health challenges (depression, helplessness, anxiety, stigmas) high levels of poverty, low levels of education minimal job opportunity, somewhat closed off community, high rates of alcohol, drugs, suicide, STIs, violence, domestic violence.” [Public health nurse, health and human services] “Homelessness in the community which relates to economics. Drugs and alcohol affect families and especially the children. Number of individuals in jail. Not enough mental health providers.” [President, multisector]
Social Justice (*N* = 12)	Themes of incarceration policies and practices were described, including convictions and the criminalization of substance use in lieu of treatment for substance use disorders; historical and generational trauma, lack of intergenerational wealth, land distribution policies that disproportionately affect indigenous communities; lack of cultural and community representation in local and county policy; structural and institutional racism and discrimination, including how these are barriers for policy goals.	“The root causes of health inequity in the community I serve is the criminalization of people who use drugs. There is also institutional racism, sexism, and other stigma tied to this. The separation of mental health disorders from substance use disorders feeds this as well.” [Programs director, multisector] “Wealth inequity and the lack of generational wealth that sustains generations. Housing market that predominantly serves college residents and the tourism market. Racism and discrimination remain a significant problem for all policy goals.” [Public affairs director, policy]
Food (*N* = 8)	When food was highlighted as a concern in leaders' communities, food deserts, access to nutritious food, food insecurity, and poor nutrition were described. Similar to other SDoH, food systems were often talked about in synchrony with other factors affecting the community’s overall wellbeing and health, such as economic opportunities and transportation.	“We serve the poorest and neediest senior over 60 and those under with a disability. These clients do not make enough money on their Social Security to afford housing, food, and health. If a client is just 5 dollars over the limit for AHCCCS [Arizona Health Care Cost Containment System, Arizona’s Medicaid program] they can't afford food.” [Client services manager, health and human services] “Low employment opportunities, vast low-income neighborhoods, diet-related disease, lack of transportation i.e.: valley-wide transit line, diminished access to healthy food with widespread food desert.” [Executive director, multisector]
Environmental Quality (*N* = 6)	This theme largely focused on a lack of basic and critical utilities such as running, drinking or wastewater, and electricity. These issues were often described in connection with economic opportunity and living in rural or tribal lands with limited access to resources compared to cities and more urban areas of the region.	“The root causes of health inequity in our community have to do with access, many of our community live in very rural areas that require travel of great distances to get access to care. There is also in the same vein a very poor population that live without even basic necessities like electricity, running water and internet service.” [County manager, policy] “Agricultural challenges due to heat & environment.” [Executive director, multisector]
Community Design (*N* = 3)	Community design as a cause of inequity was defined by lack of infrastructure, including limited land development, lack of physical infrastructure such as streets and sidewalks, and little interest in improving or developing infrastructure. A couple of examples described how insufficient physical infrastructure affects the communities served and made connections between community design and inequity.	“[My] county is a politically conservative community and there is little interest in improving and building infrastructure needed to address the environmental inequities (neighborhoods with paved and unpaved streets, no sidewalks, etc.).” [Director, health and human services] “Topography, high desert with little infrastructure. Limited land development and ownership.” [Economic development manager, multisector]

In our analysis, two themes emerged beyond the a priori SDoH, including geographic location and local political context.

##### *Geographic location (N* = *27)*

Given the rurality of northern Arizona, it was no surprise that many leaders identified rurality as a cause of inequity in their community. Participants talked about rural, remote, or isolated areas and a lack of connection as a function of rurality. For instance, rurality was considered to compound a lack of or limited access to various services and resources, such as limited healthcare services often due to long distances to travel to care, lack of affordable housing, with the most affordable housing being in more rural and isolated areas, and unfunded and underperforming schools. A few participants who discussed rurality noted the disparities between rural and urban areas in their communities, observing that rural areas experienced greater challenges compared to urban areas due to limited access to essential social services and goods.

“The disparity between rural and urban areas in the county. Lack of infrastructure: broadband, available land for private use, water, and other support utilities. These conditions negatively affect opportunities for economic development and mobility, and access to health.” [Assistant facilities management director, multisector]

“Economic disparity in rural communities across the region, combined with isolation from needed services (social, healthcare). Additionally, rural Arizona's political attitudes of self-reliance, does not provide adequate support for needy populations.” [Transit planner, transportation]

##### Political context (*N* = 10)

A number of participants voiced their thoughts on how politics play a role in health inequities. Topics commonly mentioned related to politics were lack of trust and confidence in the government, political leadership that historically and currently does not represent the community’s diversity and needs, and perceived unfair tax systems.

“Historic and continued lack of representation at the local and county level being anything other than white, male dominated.” [Senior program coordinator, multisector]

“Rural area with low education standards, underfunded and underperforming schools, and lack of economic opportunities. General apathy towards education, along with a desire to live ‘off the grid’ and away from real or perceived government intervention. Perception that taxes and government interventions are already too high.” [Assistant county manager, policy]

### Strategies to address root causes of health inequity

Approximately 63% (*n* = 130) of participants identified key strategies they considered essential in addressing health inequities in their local communities and society as a whole. Leaders described strategies that fell into ten broad categories, including 1) build community knowledge and capacity; 2) develop economic and workforce infrastructure; 3) activate collaboration and partnerships; 4) establish referral and resource systems; 5) provide direct services; 6) ensure flexible, fair, and equitable access; 7) conduct community outreach and engagement; 8) engage in advocacy and policy change; 9) be culturally and community responsive; and 10) utilize evidence-based practices (Table 4). Several strategies were oriented towards working directly with the community, such as building community capacity and engaging the community to work together towards positive change. Leaders also described strategies in response to community needs, such as establishing resource systems, directly providing needed services, and making sure access to services is flexible, equitable, and culturally grounded. Importantly, participants also discussed strategies for advancing health equity through activating partnerships, using evidence-based practices to make decisions and promote health, and engaging in advocacy to create policy and systems change.

**Table 4:** Strategies to Address Root Causes of Health Inequity (*n* = 130)

Strategies	Definition	Exemplar Quotes
Build Community Knowledge and Capacity	Provide general education and raise awareness across a range of topics; ensure appropriate framing and messaging are used when sharing information; support and equip people with tools to be successful	“Education and awareness building to support people to become their own advocates.” [Regional director, multisector] “Honest education regarding risk/benefits of chosen lifestyles that contribute to long term poor health and poor quality of life.” [Registered nurse, health and human services]
Develop Economic and Workforce Infrastructure	Develop the local economic and physical infrastructure, including employment opportunities and professional development; expand existing services; and seek funding for further development	“We have been trying to attract some different types of businesses that could employ people who have little or no secondary education.” [City manager, multisector] “Economic development efforts, development of regional transit service.” [Community development director, community and economic development]
Activate Collaboration and Partnerships	Actively search for opportunities to collaborate across organizations, sectors, and with community; build partnerships and capitalize on existing partnerships; network, share resources, align priorities, and fill gaps to achieve health equity	“Collective community collaborations, sharing of resources among community agencies, looking for avenues to partner with others.” [CEO, health and human services] “Community partnership to tackle infrastructure challenges together versus in silos. Strength is in numbers and joining forces is critical for funding and future enhancements.” [Chief information officer, other sector]
Establish Referral and Resource Systems	Connect individuals to and provide assistance in navigating needed resources and services; raise awareness of and encourage engagement in existing resources and services	“Linking people to community resources is the best strategy I see to help individuals and families address the challenges they face and find support to overcome many of the problems that occur.” [Faculty, health and human services] “The school district provides a full time RN to services our students. She provides referrals as needed.” [Superintendent, education]
Provide Direct Services	Address health equity by directly providing services and resources that respond to community needs	“Delivery of services which are responsive to these challenges.” [Director, health and human services] “Provide as much food as possible so no one goes hungry.” [Soup kitchen supervisor, food systems]
Ensure Flexible, Fair, and Equitable Access	Provide services and resources to everybody regardless of financial or other barriers; ensure resources and services are free of cost or low cost and accessible; meet people where they are by providing services where they are needed	“Meeting clients where they are. Coming to them.” [Health educator, food systems] “Providing services to all regardless of income or ability to pay; hiring compassionate, non-judgmental, knowledgeable service providers.” [Division manager, health and human services]
Conduct Community Outreach and Engagement	Have an active presence in the community, build rapport and trust with communities, conduct outreach via various communication means to connect with the community, listen and respond to the need, and learn from the community to work together toward health equity	“Putting a 'face' to local government–helping residents see that public servants are not part of a nameless machine, rather they are friends, neighbors and live in the same communities.” [Assistant county manager, policy] “Work with positive community members that want to help students, participate in local radio show in the past to give positive messages, newsletters, open listening, focus decisions on what is best for students, try and recruit positive role models for children.” [Superintendent, education]
Engage in Advocacy and Policy Change	Advocate and lobby for resources, services, and policy changes based on community needs; raise awareness of locally-identified issues and influence decision-makers to take action	“Provider groups banding together to lobby for change.” [CEO, multisector] “Advocating for system review/change. Push for outcomes vs outputs. Asking 3 questions: How much did you do, how well did you do it and is anyone better off?” [Director and chief health officer, multisector]
Be Culturally and Community Responsive	Recognize and honor the uniqueness of different cultures and communities; provide services and resources that are grounded in the culture and community	“Our organization tries to bring together professionals from a range of sectors, help ensure that prevention strategies are culturally, linguistically, and age appropriate, and that they match people’s health literacy skills, provide internet skill-building courses to help residents find reliable prevention services.” [Executive director, multisector] “Acknowledgment of historical trauma and focus on resiliency building for children and youth.” [Executive director, multisector]
Utilize Evidence-Based Practices	Stay informed on and implement evidence-based practices into strategies used to address health equity	“Being informed on evidence-based practices and incorporating them into our strategies. Updating policies to prioritize addressing root causes, rather than how we ‘feel’ about them.” [Chief probation officer, multisector] “Working with community residents and partners, achieving agreement on proposed service delivery models, implementing evidence-based programs, and monitoring/providing feedback on program results. When supported, adopt public health ordinances to promote health (i.e., smoking ordinances, texting while driving ordinances, etc.).” [Deputy director, health and human services]

## Discussion

The goal of the RHES was to understand multisectoral leaders’ perspectives and strategies for action on the social determinants of health and the root causes of health inequity in the largely rural, culturally diverse region of northern Arizona. Specifically, the RHES assessed knowledge, attitudes, and actions among 206 county-level leaders representing five counties and 13 distinct sectors. We found multisectoral leaders varied in their understanding of the SDoH and the root social, economic, and environmental causes of health inequity experienced by their communities and were encouraged by the creative community-focused strategies to address health inequities locally and regionally.

Although leaders were provided the definition and asked to describe the root causes of inequity, which are defined by elements of interlocking systems of injustice and oppression, the majority of leaders instead provided concrete examples of SDoH. Although an important step in a common language across differing sectors, the SDoH framework does not critically analyze or address the underlying social, economic, and environmental conditions that produce inequities generally and health inequity specifically [5, 25]. When participants from different sectors were aware of the drivers of health inequity and were especially cognizant of the SDoH facing the communities they serve, they clearly articulated the interplay of complex systems of oppression that place people of color and individuals living in poverty at a greater disadvantage and how this disadvantage can lead to adverse health outcomes in their communities. Among these leaders, and despite the variability in how actors from different sectors understand the concept of health inequity, a desire to change the status quo was apparent. Ultimately, our findings suggest that although multisectoral leaders recognize SDoH and to some extent the root causes of health inequity, and are motivated to collaborate to create positive change, they may not have a common understanding of what health equity is and therefore, how to act to advance health equity through policy, program, and practice goals. While professional differences in training and approach may support the holistic understanding of a topic (i.e., different sectors can leverage their knowledge, skills, and resources and tackle the issue from various angles), a shared vision of health equity is critical for each sector to be able to leverage their expertise and collaborate meaningfully and effectively.

Our work is consistent with previous findings that describe the lack of a universal understanding of health equity [5, 25, 26], especially when considering perspectives from across disciplines and sectors, participating leaders had differing understandings of the root causes of health inequity. Without a common language for health equity, creating and sustaining multisectoral partnerships as well as guiding policy and resource allocation, while remaining respectful of populations of focus, can be limited [17, 18, 25]. The root causes of health inequity are diverse, complex, dynamic, and interdependent [19], making clarity and intentionality of utmost importance when pursuing equity efforts that strategically involve various stakeholders with their own agendas [5]. Without a clear consensus on health equity, stakeholders often struggle to agree on concrete goals and conditions of success potentially contributing to co-opted or wasted resources and efforts and initiatives that lose their focus on health equity [5, 25]. Having a common language and shared vision for health equity across sectors can contribute to developing multisectoral partnerships and that influence the SDoH and the larger social and economic environment that determine the health and wellbeing of marginalized populations [15, 17, 18, 25].

Furthermore, leaders identified strategies across ten broad areas to address the challenges their communities experience. These suggested strategies indicate leaders are aware of health inequities and their drivers, and they are well-positioned to create and implement community-oriented solutions. Leaders expressed value in community partnerships and multisectoral collaborations to develop and advance health equity initiatives. These responses are supported by the literature and the multiple benefits, including pooling resources, leveraging unique knowledge bases, expanding reach, and avoiding duplication of work a multisectoral collaboration can have [27]. Leaders also recognized that multisectoral action can help address health inequity because it recognizes that the social and economic factors that influence health often lie outside of the domain of the health sector [28]. For example, multisectoral collaborations show promise in developing supportive environments that could enhance access to essential services for marginalized populations [28]. Despite challenges to developing successful multisectoral collaborations, recent research by Narain et al. [18] has shown that framing health equity issues in ways that resonated with sectors outside of public health was valuable for promoting work across sectors to improve health equity. This includes, for example, using more inclusive language that is understood across sectors, aligning priorities including missions and operational costs, and creating a shared vision with partners and community stakeholders. Furthermore, highlighting how health equity goals advance the missions of sectors outside of health services and public health helps foster support for health equity in these other sectors and develop more effective collaborations [18]. This finding further supports the need for a common lexicon and shared values in creating and sustaining multisectoral collaborations determined to address health equity.

### Implications for public health practice and research

Our findings indicate that multisectoral leaders in northern Arizona recognize the SDoH in the communities they serve. However, recognition alone is insufficient to improve health equity. The next step is action, embracing a multisectoral approach to engage broad stakeholders, including private, public, and grassroots organizations and community members in addressing health inequities. Leaders can look at existing efforts and use creativity and innovation to engage the community and multisectoral stakeholders in building equity solutions together. The Bay Area Regional Health Inequities Initiative is an example of effective cross-sectional work with 10-member health departments and over 200 community partners collaborating to drive programmatic, systems, and policy change that enables healthy communities and economic prosperity for all in the California Bay Area [29].

Similarly, before action, there is a need for shared language and practice on the concept of health equity. Previous research highlights the importance of a common language in conducting health equity work as well as developing and maintaining partnerships across sectors to solve inequity issues together [17, 18, 25]. The advancement of health equity and the elimination of social-structural inequities also require the engagement of critical epistemology and praxis that decentralize health research and institutions as the only routes to achieve health equity [30]. Historically oppressed communities, policymakers, stakeholders, and public health researchers are at the frontlines in ensuring that the crucial elements of health equity are understood in public and private sectors [25], and thus, should set the stage for a common language when engaging in multisectoral work. This could be achieved, for instance, by including a clear definition with essential values of health equity in state health improvement plans. Finally, the COVID-19 pandemic has shown a spotlight on longstanding inequities in the US and globally with its disparate impact on marginalized communities. Health equity frameworks and multisectoral approaches will be essential in alleviating the impacts of the pandemic on the SDoH for already disadvantaged populations, saving lives, and advancing health equity overall [31, 32].

Health inequity is driven by unequal allocation of power and resources, which in turn manifests in the SDoH impacting individual and community health [3]. A multisectoral approach to health equity may offer opportunities to begin dismantling power imbalances by facilitating community empowerment and capacity building – such as through partnership knowledge and power-sharing and the community’s involvement in priority setting and decision-making [33]. Although not a focus of this study, future research may explore multisectoral collaboration as a way to evaluate and create equitable power relations between community and multisectoral leaders, build capacity in communities to advocate for health equity solutions, and yield power back to those who are most affected by inequity.

As a participant in the NIH Research Centers in Minority Institutions (RCMI) Program which aims to “develop and strengthen the research infrastructure necessary to conduct state-of-the-art biomedical research and foster the next generation of researchers from underrepresented populations,” our Southwest Health Equity Research Collaborative (SHERC) leveraged the RHES to inform the focus of our Center’s community engagement and investment in research infrastructure. By engaging multisectoral leaders across our region, we as a university partner are now in a better position to understand from a SDoH and health equity frame the community-identified research issues and solutions that our Center could mobilize to address as a regional research partner. As a result of the RHES, several local new community partnerships have been formed to engage community voices on the topic of health equity. In one project, we are pursuing a photovoice methodology to understand community perceptions on COVID-19 health inequity in a northern Arizona county. In another, we are employing a collective impact model aimed at enhancing the children's health system in a different regional county.

As an RCMI, we have taken intentional steps to structure our internal research grant opportunities to support the strategies to address the root causes of health equity outlined in Table 4. Through our community engagement, research infrastructure, and investigator development cores, we have also outlined several intentional steps to reflect community needs in our research training, technology, and targeted funding initiatives. These steps will ensure we are growing a research infrastructure that aligns with the ‘on the ground’ issues facing multisectoral leaders in our region and leverages the full interdisciplinary and team science approach required to solve complex public health problems of this century.

### Limitations and strengths

Based on standard procedures used in qualitative research studies, purposive sampling methods were used to recruit multisectoral leaders from the northern Arizona region [34, 35]. While purposive sampling methods are highly vulnerable to selection bias and sampling error, recent research has shown that these methods are more efficient and result in more ‘information-rich’ cases for qualitative inquiry [36, 37].

Data adequacy in qualitative research is determined by both the appropriateness of sample size and sample composition [38]. Using a community-engaged approach to recruit participants for the RHES ensured that respondents were recognized as sector leaders in their community. While we successfully obtained representation from all 13 sectors, 95% of all participants identified with either health and human services (49%), education (26%), or community and economic development (20%). This imbalance in sample composition is likely due to individuals who identified with more than one sector, as is often the case in less populated counties where individuals may be responsible for leading more than one department.

We also acknowledge the lack of racial and ethnic diversity in our respondents. Still, we are uncertain if this is a limitation of our recruitment strategy or a true reflection of the lack of diversity among leadership in northern Arizona. In an effort to obtain a sample representative of county leadership, we engaged county champions and members of the CAC in the development of participant lists. We presented survey results back to champions to interpret this lack of diversity in the sample, which is not representative of the racial and ethnic composition of the counties and communities served. Generally, this lack of diversity was interpreted as a true reflection of the composition of the leadership and an opportunity to critically reflect on how to improve racial and ethnic diversity and community representation within these leadership positions.

Moreover, the lack of diversity in our sample aligns with recent studies that found that the US public health workforce employed at local, state, and federal health departments is largely non-Hispanic white [39]. Underrepresentation of people of color in supervisory and managerial roles is even more pronounced [39, 40]. Fewer than 10% of top executives at local health departments nationally identify with a race other than white, a figure that has stayed low since 2008 [41]. Workforces that are more diverse and representative of the communities they serve are better positioned to advance health equity in those communities through creative and culturally ground problem-solving [39, 40]. This highlights the need to prioritize efforts to cultivate multisectoral leadership diversity both locally and nationally.

With a completion rate above 60% and participation across all sectors and counties of interest, including many sectors not typically included in equity work, we are confident that the outcomes of the RHES capture the perspectives of multisectoral leadership in the northern Arizona region. This research was completed prior to the COVID-19 pandemic, which has potentially created more awareness about health equity for those in sectors outside of the health services or public health sectors. Additional assessment post COVID-19 of health equity among multisectoral representatives is necessary.

## Conclusions

Numerous health inequities and their drivers were identified by community leaders from different sectors in the northern Arizona region. Discrimination and disproportionate allocation of power and resources were listed as common root causes of health inequity. Many of the respondents also recognized the link between the SDoH and the existing health inequities. A majority of responses (75%) were categorized as SDoH across sectors, where leaders often described complex synergies between the various factors and systems impacting the communities they serve. Community leaders in the northern Arizona region acknowledge the importance of multisectoral partnerships and collaborations in improving health equity for their communities but a common understanding of health inequities remains to be widely established and is essential for conducting effective multisectoral work. This baseline assessment will serve as the basis for a productive dialogue about the various and unique contributions that each sector can activate to influence and strengthen health equity in our region.

## Supplementary Information

**Additional file 1.** NAU-SHERC Regional Health Equity Survey, Data Collection Instrument. Description of data: Online survey instrument used to collect data for the Regional Health Equity Survey

## Data Availability

The datasets generated during and/or analyzed during the current study are not publicly available but are available from the corresponding author on reasonable request.

## Abbreviations

CAC: Community advisory council
CEC: Community engagement core
BARHII: Bay Area Regional Health Inequities Initiative
RCMI: Research Centers in Minority Institutions
RHES: Regional Health Equity Survey
SDoH: Social determinants of health
SHERC: Southwest Health Equity Research Collaborative
