# Supplementary material for: A multisectoral approach to advance health equity in rural northern Arizona: county-level leaders’ perspectives on health equity
Source: BMC Public Health. 2022 May 13;22:960. doi: 10.1186/s12889-022-13279-6 (PMC9100312; doi:10.1186/s12889-022-13279-6)
Supplement: Supplementary file 1 — Additional file 1. NAU-SHERC Regional Health Equity Survey, Data Collection Instrument. Description of data: Online survey instrument used to collect data for the Regional Health Equity Survey [file 12889_2022_13279_MOESM1_ESM.pdf]

NAU
NORTHERN ARIZONA UNIVERSITY

INTRODUCTION

Regional Health Equity Survey

You are being asked to participate in this 20-minute survey because you have been identified as a leader in your field which may include health, housing, transportation, planning, justice, grassroots organizing, and policy among many other areas important to community well-being. We at the Northern Arizona University (NAU), Center for Health Equity Research (CHER) will survey approximately 300 community, organizational, and university leaders from across 7 counties and 14 Native Nations in Arizona.

We aim to assess our regional and collective capacity to address the root causes of health inequity. Health inequities are caused by the underlying social inequalities which create different conditions in which people are born, grow, live, work, and age.

Participation is voluntary and all individual responses will remain confidential. You will receive a $10 gift card upon completion of the survey. You may skip any question you do not want to answer. You may complete the survey in multiple sessions. Use the buttons at the bottom of the survey to navigate through the survey. Do not use your browser's back button.

Benefits to participants:

- **Establish a baseline of Northern Arizona counties and Native Nations organizational capacity to address health equity.**
- **Develop county- or Native Nation-level reports to advance collective impact strategic planning.**

- **NAU-CHER learns how to best support regional capacity to impact health inequity through tailored research/evaluation, advocacy, policy, and workforce development and training.**

We thank you for your time and expertise.

For questions please contact:
Mark Remiker
Program Coordinator, Sr.
Northern Arizona University
Center for Health Equity Research
mark.remiker@nau.edu
(928) 523-4295

Funding supported by NIH/NIHMD RCMI U54MD012388

PERSONAL

Which best describes the primary organization you work for?

- ○ County agency
- ○ Tribal agency
- ○ State-level agency
- ○ Federal agency
- ○ Faith-based organization
- ○ Academic institution
- ○ Private sector business
- ○ Community-based organization
- ○ Community group/coalition
- ○ Other __________

What county do you work for? If you work for more than one county, please list the primary.

- ○ Apache
- ○ Coconino
- ○ Mohave
- ○ Navajo

- Yavapai
- Yuma
- ______ Other

What tribe do you work for? If you work for more than one tribe, please list the primary.

- Cocopah
- Ft. Mojave
- Havasupai
- Hopi
- Hualapai
- Kaibab Paiute
- Navajo
- Quechan
- San Carlos Apache
- San Juan Southern Paiute
- Tonto Apache
- White Mountain Apache
- Yavapai Apache
- Yavapai Prescott
- Other ______

With which sector/area does your work most closely align? *check all that apply*

- Community and economic development
- Health and human services
- Law, justice, and public safety
- Parks and recreation
- Policy
- Early childhood development
- Transportation
- Food systems
- Housing
- Education
- Arts, music, and culture
- Planning and zoning
- Cultural resources management and preservation
- Other ______

What is your current position or title?

______

How long have you been in your current position? (Please enter the number of months only if it has been less than one year. Otherwise, answer in years only.)

Months

Years

How long have you worked in your field or sector? (Please enter the number of months only if it has been less than one year. Otherwise, answer in years only.)

Months

Years

How would you describe the primary community you serve?

What is your primary service area? (e.g., city, county, district, region, etc.)

Do you work directly with community residents in your current position?

- Yes
- No

Do you supervise staff members who work directly with community residents?

- Yes
- No

DEFINITIONS

Throughout this assessment, we will use terms related to health inequity that may be unfamiliar to you. By clicking on the link below, you will open up a new window that includes written definitions and examples of some of these terms. If needed, you can leave this window open and refer to these definitions as you take the survey.

SURVEY DEFINITIONS

COMMUNITY

The next set of questions will ask about the well being of the community you serve.

To begin, what are the **root causes** of health inequity, or the primary social, environmental, or economic conditions that impact the community you serve?

In your position, what strategies have you found to be successful in overcoming these challenges?

How uneven is the distribution of these resources and services in the community you serve?

	Very uneven	Somewhat uneven	Not at all uneven
Legal/justice services	◯	◯	◯
Health care coverage	◯	◯	◯
Adult education	◯	◯	◯
Mental health services	◯	◯	◯
Children's education	◯	◯	◯
Employment	◯	◯	◯
	Very uneven	Somewhat uneven	Not at all uneven
Substance abuse services	◯	◯	◯
Food security	◯	◯	◯
Civic engagement	◯	◯	◯
Relationships and social support	◯	◯	◯
Income	◯	◯	◯
Childcare	◯	◯	◯
	Very uneven	Somewhat uneven	Not at all uneven
Housing	◯	◯	◯
Mobility/transportation	◯	◯	◯
Public safety	◯	◯	◯
Disability services	◯	◯	◯
Power	◯	◯	◯
Wealth	◯	◯	◯
	Very uneven	Somewhat uneven	Not at all uneven
Primary care services	◯	◯	◯

	Very uneven	Somewhat uneven	Not at all uneven
Technology/internet	◯	◯	◯
Other ______	◯	◯	◯

ORGANIZATION

This section of questions relates to how your organizational culture and strategic planning processes focus on addressing the root causes of inequity in community you serve.

Click here for definitions

In your opinion, how much does your organization focus on addressing the **root causes of inequity** in the community you serve?

- ◯ There is no focus on the root causes of inequity.
- ◯ There is not enough focus on the root causes of inequity.
- ◯ There is about the right amount of focus on the root causes of inequity.
- ◯ There is too much focus on the root causes of inequity.
- ◯ I don't know.

Does your organization's strategic plan include a commitment to addressing the **root causes of inequity** in the community you serve?

- ◯ Yes
- ◯ No
- ◯ I don't know whether the strategic plan addresses the root causes of inequity.
- ◯ I don't know whether there is a strategic plan for my organization.
- ◯ Not applicable: There is not a strategic plan for my organization.

Does your organization's mission statement express a commitment to addressing the **root causes of inequity** in the community you serve?

- ◯ Yes
- ◯ No
- ◯ I don't know whether the mission statement addresses the root causes of inequity.
- ◯ I don't know whether my organization has a mission statement.
- ◯ Not applicable: There is not a mission statement for my organization.

For each of the following statements, please indicate the response that most closely describes your organization.

My organization...

	No	Moving in that direction	Yes	Don't know
Makes deliberate efforts to build the leadership capacity of community members to advocate on issues affecting the environmental, social, and economic conditions.	◯	◯	◯	◯
Demonstrates a commitment to addressing environmental, social, and economic conditions that impact health.	◯	◯	◯	◯
Has strategies in place to minimize barriers to community participation (e.g., it is possible to provide money for child care and transportation to residents attending community meetings, etc.).	◯	◯	◯	◯

	No	Moving in that direction	Yes	Don't know
Uses data to make decisions about how to distribute resources and services within the community we serve.	◯	◯	◯	◯

	No	Moving in that direction	Yes	Don't know
Demonstrates a commitment to working with external partners, policy-makers, and community members to address environmental, social, and economic conditions.	○	○	○	○
Has strategies in place to advocate for public policies that address environmental, social, and/or economic conditions.	○	○	○	○

In your current position, have you ever received training about the different ways to address environmental, social, and economic conditions that impact the community you serve?

- ○ Yes
- ○ No
- ○ I don't remember

What is the name of the training you received?

How useful was your training in helping you address environmental, social, and economic conditions that impact the community you serve?

- ○ Very useful
- ○ Somewhat useful
- ○ Neither useful nor useless
- ○ Somewhat useless
- ○ Very useless

What role do you have in making decisions that affect your organization's efforts to address the root causes of inequity n the community you serve?

- ◯ I have no decision-making role.
- ◯ There are opportunities for me to give input, but I don't have a role in seeing that my input is incorporated into the decision.
- ◯ I have an active role in major decisions affecting my organization's efforts to address the root causes of inequity in the community we serve.
- ◯ I have primary decision-making power for my organization.
- ◯ Addressing the root causes of inequity in the community we serve is not a focus of my organization.
- ◯ [ ] Other

EXPERIENCES

For the next set of questions we are interested in learning about your personal knowledge and experience related to the social determinants of health which include aspects of the environmental, social, and economic conditions that impact health.

Click here for definitions

Please indicate how much you agree or disagree with the following statements:

	Strongly disagree	Disagree	Neutral	Agree	Strongly agree
Being aware of my own beliefs, values, and privilege helps me understand others' perspectives.	◯	◯	◯	◯	◯
Staff I interact with at my organization are comfortable talking about race and racism	◯	◯	◯	◯	◯

Staff I interact with at my

	Strongly disagree	Disagree	Neutral	Agree	Strongly agree
Staff I interact with at my organization are comfortable talking about discrimination.	○	○	○	○	○
I believe it is important to understand the beliefs and values of the residents and community members that I serve.	○	○	○	○	○
I understand what the environmental, social, and economic conditions are that impact health.	○	○	○	○	○
I regularly have meaningful interactions and have learned from people of different cultures and backgrounds from my own.	○	○	○	○	○
Senior management at my organization are comfortable talking about discrimination.	○	○	○	○	○
I have taken steps to enhance my own cultural humility, cultural competence, and/or cultural understanding (for example, through trainings, self-reflection, personal relationships, etc).	○	○	○	○	○
I could explain the environmental, social, and economic conditions that impact health to my co-workers.	○	○	○	○	○
I work with a culturally diverse staff.	○	○	○	○	○
In general, my organization's programs are structured to address the environmental, social, and economic inequalities in our community.	○	○	○	○	○
Senior management at my organization are comfortable talking about race and racism.	○	○	○	○	○

Please indicate how much you agree or disagree with the following statements about the opportunities you have to reflect on addressing health inequities in your work:

Strongly Strongly

	Strongly disagree	Disagree	Neutral	Agree	Strongly agree
I subscribe to a listserv, online discussion group, e-mail list, or other web-based source for learning about developments on the topic of health inequities on an ongoing basis.	○	○	○	○	○
Staff are encouraged to learn about ways to address the environmental, social, and economic conditions that impact health **from one another**.	○	○	○	○	○
Staff are encouraged to learn about ways to address the environmental, social, and economic conditions that impact health **from external sources**.	○	○	○	○	○
Within my organization, we have engaged in group discussions about how our work could address one or more of the environmental, social, and economic conditions that impact health.	○	○	○	○	○

PARTNERSHIPS

The next section will ask you about the extent that your organization collaborates with other public agencies, institutions, and community-based organizations on the **root causes of health inequities.**

Click here for definitions

To what extent does your organization partner with public agencies, institutions, or community-based organizations on the following issues?

	None	Some	A lot
Food security	○	○	○

	None	Some	A lot
Land-use planning	○	○	○
Community safety and violence prevention	○	○	○
Early childhood development and education	○	○	○
Transportation	○	○	○
	None	Some	A lot
Community economic development (e.g., job creation, business development, etc.)	○	○	○
Availability of quality affordable housing	○	○	○
Racial justice	○	○	○
Quality public education	○	○	○
Environmental justice	○	○	○
	None	Some	A lot
Arts and culture	○	○	○
Recreation opportunities, parks, and open space	○	○	○
Youth development and leadership	○	○	○

In your current position, please indicate which of the following **SECTORS** you have partnered with in the past and which **SECTORS** you would like to partner with in the future to address the environmental, social, and economic conditions that impact health.

	In the **PAST**, I have partnered with the following **SECTORS**<br><br>Check all that apply	In the **FUTURE**, I would like to partner with the following **SECTORS**<br><br>Check all that apply
Cultural resources management and preservation	☐	☐
Health and human services	☐	☐
Parks and recreation	☐	☐
Education	☐	☐
Arts, music, and culture	☐	☐

	In the **PAST**, I have partnered with the following **SECTORS**<br><br>Check all that apply	In the **FUTURE**, I would like to partner with the following **SECTORS**<br><br>Check all that apply
Housing	☐	☐
Policy	☐	☐
Early childhood development	☐	☐
	Check all that apply	Check all that apply
Planning and zoning	☐	☐
Law, justice, and public safety	☐	☐
Food systems	☐	☐
Transportation	☐	☐
Community and economic development	☐	☐
Other ________	☐	☐
Other ________	☐	☐

Please tell us why you'd like to partner with the sectors you listed above.

____

In your experience, what are **the three most important** elements for developing a successful partnership internal or external to your organization?

- ☐ Recognition of different kinds of expertise
- ☐ Accountability/follow-though
- ☐ Transparency
- ☐ Ability to resolve conflict
- ☐ Communication
- ☐ Shared decision making
- ☐ Inclusion
- ☐ Humility
- ☐ Trust
- ☐ Reciprocity
- ☐ Shared vision
- ☐ Other ________

DATA

Next, you'll be asked questions about how you use data to make decisions

In your position, do you use data to make decisions about the projects you work on?

- Yes
- No

What types of data do you typically use to guide your work and/or make decisions?

Ideally, how often would you use data to guide your decision making?

- Never
- Rarely
- Sometimes
- Often
- Always

In reality, how often are you able to use data to make decisions?

- Never
- Rarely
- Sometimes
- Often
- Always

Do you have any barriers to using data to make decisions?

- Yes
- No

What are the biggest barriers to using data? *check all that apply*

- [ ] Lack of support from leadership
- [ ] I do not have time
- [ ] Useful data is available, but is outdated
- [ ] I do not have access to data
- [ ] No staff with expertise in analysis
- [ ] Useful data does not exist
- [ ] ________ Other

INITIATIVES

Next we would like to ask you about projects or initiatives in your field that inspire you. Examples can be real and ongoing in your community or state or national examples you would like to see happen in your community. Please feel free to either type in examples, paste links to websites, or upload reports.

Please paste links or type in examples here

Upload examples here

In your opinion, what made these initiatives successful?

RESEARCH

What role do you think research has in addressing the environmental, social, and economic conditions that impact health in the community you serve?

In what areas of research could NAU concentrate support in order to best improve health inequities in your community?

Please tell us how concentrating support in these areas would improve health inequalities in your community.

DEMOGRAPHICS

You're almost done! We just need to ask you a few more quick questions about yourself. Since we'll be asking you to participate in a follow-up survey in the next few months, we will be asking you to provide us with your name and e-mail address. This information will only be used by our staff. All of your answers will still remain confidential.

What is your age?

What is your gender identity?

- Male
- Female
- Agender/nonbinary
- Prefer not to answer
- Other

What is your race and ethnicity? *check all that apply*

- Black/African American
- American Indian/Alaska Native
- Hispanic/Latino(a)
- White
- Asian/Pacific Islander

- ○ Prefer not to answer
- ○ ______ Other

What is your tribal affiliation?

- ○ Cocopah
- ○ Ft. Mohave
- ○ Havasupai
- ○ Hopi
- ○ Hualapai
- ○ Kaibab Paiute
- ○ Navajo
- ○ Quechan
- ○ San Carlos Apache
- ○ San Juan Southern Paiute
- ○ Tonto Apache
- ○ White Mountain Apache
- ○ Yavapai Apache
- ○ Yavapai Prescott
- ○ ______ Other

What county do you live in?

- ○ Apache
- ○ Coconino
- ○ Mohave
- ○ Navajo
- ○ Yavapai
- ○ Yuma
- ○ ______ Other

What city or town do you live in?

Would you be interested in sharing your thoughts about health equity in your community on an NAU health equity podcast?

- Yes
- No

If there is anything else related to the health and well-being of your community that you'd like to address, please share it with us in the space provided below.

THANK YOU

We thank you for taking the time to complete this survey.

To submit your responses, please click "SUBMIT SURVEY".

Powered by Qualtrics
